# 

**Published:** 1891-02-28

**Authors:** 


[Registered at the General Post Office as a Newspaper for transmission in the United Kingdom and Abroad.]

[illegible]

Per Annum, 6s. 6d.; post free.

Monthly Parts, 6d.; post free 8d.

Ditto per Annum, 8s., post free.

### THE HOSPITAL

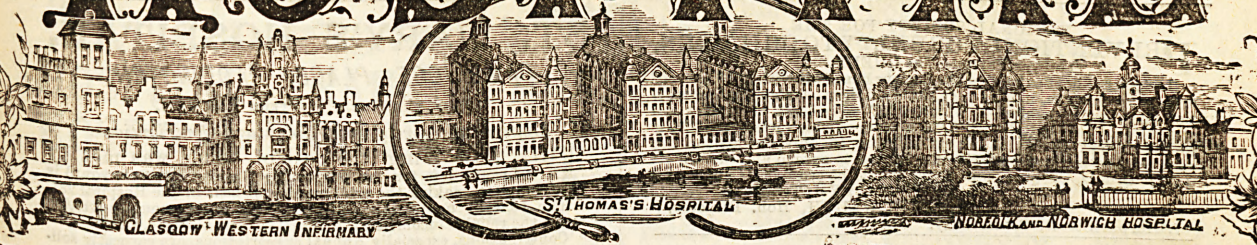

A WEEKLY JOURNAL OF

### Science, Medicine, Nursing and Philanthropy.

### SATURDAY, FEBRUARY 28th, 1891.

**The Doctor's Difficulties in Town and Country** - 319
**Passing Topics.**—Give and Take - 320
**The Doctor's Armchair.**—The Other Side of the Shield - 321
**Everybody's Page** - 322
**The Microscope in Medicine.** By FRANK J. WETHERED, M.D.—VII. Methods of Staining Sections of the Nervous System - 323
**Notes and News** - 324
**General Charities** - 325
**Annotations.**—The Seamen's Hospital—Inoculation for Crime—Women's Greatest Right - 326
**Scraps and Gleanings** - 325
**The Practitioner's Mirror and Retrospect:—**
MEDICINE.—Gastric Ulcer - 327
SURGERY.—Antiseptic Surgery - 328
THERAPEUTICS.—Escholtzia Californica - 329
OPHTHALMATOLOGY.—Granular Ophthalmia - 329
NEW DRUGS, APPLIANCES, AND THINGS MEDICAL.—"Coralline" Food - 330
**The Student of Medicine.**—Leading Athletes—Appointments—Vacancies—Pass Lists—Events for the Month of March.

### EXTRA SUPPLEMENT—THE NURSING MIRROR.

**En Passant.**—Bedfordshire Institute—The Sisters of St. John—Hampshire Nurses' Institute—A New Society—Short Items—A Woman's Life - cxix
**Lectures on Surgical Ward Work and Nursing.** By ALEX. MILES, M.B. Edin., C.M., F.R.C.S.E. Lecture XV. Dressing Trays and Anæsthetics - cxx
**Princess Christian's Daughter** - cxxi
**Presentations** - cxxi
**A Winter's Holiday.** IV. In Spain - cxxi
**Notes from Australia** - cxxii
**Examination Questions** - cxxii
**Everybody's Opinion.**—The Death Rate in Typhoid—Nurses' Home, Truro—A Source of Danger - cxxiii
**Notes and Queries** - cxxiii
**Off Duty.**—Nurse Hilary (*continued*) - cxxiv
**Appointment—Amusements and Relaxation** - cxxiv

